# Factors that Master of Nursing students associate with study progress in higher education institutions in Gauteng province

**DOI:** 10.4102/hsag.v27i0.1671

**Published:** 2022-01-31

**Authors:** Patricia Y. Mudzi, Priscilla M. Jiyane, Nombulelo Sepeng

**Affiliations:** 1 Department of Nursing, Faculty of Health Sciences, University of Pretoria, Pretoria, South Africa

**Keywords:** postgraduate, progress, research conceptions, researcher, supervisory, support, work–life, writing

## Abstract

**Background:**

Globally, the unsatisfactory progress of postgraduate students registered for a master’s degree is a cause for concern. It affects graduation numbers and completion time.

**Aim:**

This study aimed to determine the perceived supervisory-researcher community support, research writing, work–life balance, and research conceptions factors that Master of Nursing students associate with study progress.

**Setting:**

The study was conducted in three selected higher education institutions (HEIs) in the Gauteng province.

**Method:**

A correlational cross-sectional research design was utilised. A self-administered questionnaire adapted from the Cross-Country Doctoral Experience Survey was e-mailed to a total sample of 136 Master of Nursing students who were at least in their second year of study, of which 122 (89.7%) responded. Descriptive statistics, factor analysis, the Mann-Whitney U test, and the Kruskal-Wallis test were used to analyse the responses.

**Results:**

Most of the students’ perception was greater regarding supervisory-researcher community support and research conceptions. Despite a high mean composite score of 4.134 (SD-1.452) on work–life balance, some respondents found it difficult to balance work and life. Respondents with a source of income perceived greater supervisory-researcher community support (*p* = 0.022) while those studying full-time had better research writing perceptions (*p* = 0.002).

**Conclusion:**

There is need for HEIs to develop or strengthen interventions targeting research writing and work–life balance factors that were perceived to result in less support. Funding remains a concern for Master of Nursing students.

**Contribution:**

This study contributes to knowledge on factors that Master of Nursing students associated with study progress.

## Introduction

The unsatisfactory progress of postgraduate students registered for a master’s degree has become a global concern (Havenga & Sengane 2018:1). Higher education institutions (HEIs) have been awakened to the reality that turning a blind eye to this growing scourge is no longer an option (Botha 2017:342). Many factors may impact study progression in HEIs, and these factors include: supervisory-researcher community support, research writing perceptions, work–life balance, and research conceptions (Pyhältö et al. 2018:8–24). A significant amount of research has been done on factors that postgraduate nursing students associate with study progress but has largely focused on Doctor of Philosophy (PhD) students.

Despite intensified enrolments of postgraduate students (master’s students included), countries like the United States of America (US) have high attrition rates of 47% – 50% attributed to unpreparedness, poor progress, and the demanding curriculum (Xu & Grant 2018:449). In some Sub-Saharan African (SSA) countries like Nigeria, the attrition rate of Master of Nursing students was found to be 10% – 20%, and was attributed to factors such as being a full-time student and full-time worker, work–life balance as well as family issues. Master’s students were also observed to be heavily dependent on supervisor support for progression. (Onwe 2018:247). The repeated changing of supervisors was also found to impede study progress (DHET 2017:138). It is therefore important to consider factors that postgraduate nursing students associate with study progress.

Supervisory-researcher community support relates to the support that students receive from supervisors and other members of the research community (Vekkaila et al. 2018:1439). It involves support such as empathy, trust, listening, caring, advice, feedback, affirmation, and problem-solving capabilities (Pyhältö 2018:212). The support enables the creation of a relationship that allows the postgraduate student to fully participate in life’s opportunities for academic growth, research, and development that promotes study progress (Feeney & Collins 2015:113).

The supervisor and postgraduate student interaction can enable the postgraduate student to feel valued and to view difficulties in research as possibilities for growth instead of obstacles that might impede study progress (Havenga & Sengane 2018:1). An open and trusting relationship between supervisors and master’s students provides leeway to openly discuss personal matters that affected their study progress (Hajihosseini et al. 2018:98). Supervisor experience may play a pivotal role in the postgraduate student’s progress. On the contrary, a novice supervisor’s intentions to promote their development in research may conflict with strategies that foster a rich learning experience for students (Bruce & Stoodley 2013:1).

Summers and Mpanda (2014:129) noted that studying full-time resulted in 100% completion rates compared to 35.5% amongst those studying part-time because full-time postgraduate students had better access to supervisors. In some cases, postgraduate students may expect their supervisors to continuously contribute and actively participate in their research projects, whilst the supervisors may anticipate students to undertake research more independently resulting in conflicting roles (Yarwood‐Ross & Haigh 2014:38). Consequently, the postgraduate students may end up perceiving supervisors as unsupportive, unapproachable, and rigid (Havenga & Sengane 2018:1), thus hampering their study progress.

Researcher community support in the form of peer-based support promotes good interpersonal and interaction skills because of opportunities for learning working life skills (Kaakinen et al. 2016:22). Equally important is the family of the postgraduate student which provides socio-emotional support, advice, encouragement, caring, and sympathy as an integral form of researcher community support (Devaney 2015:213).

Perfectionism is a research writing perception that refers to continuously working and re-working on the material until it is free from mistakes and flaws or the student consequently gives up the effort (Pyhältö et al. 2018:14; Rahman et al. 2019:643). Perfectionism can result in postgraduate students having feelings of failure and the subsequent psychological distress slowing study progress (Hill et al. 2004:80).

Blocks and procrastination were observed to be writing perceptions that impeded postgraduate students’ study progress (Pyhältö et al. 2018:14). Blocks are the inability to produce texts and procrastination includes a trend of postponing or failure to commence tasks that promote study progress (Sala-Bubaré et al. 2018:327). Both blocks and procrastination were related to lower productivity and slowed progress (Castelló, McAlpine & Pyhältö 2017:1108).

Work–life balance refers to the degree to which an individual in higher education effectively manages multiple responsibilities at work, at home, and in other aspects of life (Atibuni et al. 2020:115). According to O’Mahony and Jeske (2019:63), master’s students experience a mix of anxiety and guilt if they are unable to support their families whilst studying leading to social withdrawal and isolation which negatively impacts their study progress.

Research conceptions focus on what carrying out research means to postgraduate students (Pyhältö et al. 2018:14). Ross et al. (2017:73) noted that some of the research conceptions master’s students had about research included their consideration of research as an area that is only meant for academic experts and not what the students may engage in through their daily professional and personal lives. According to Healey and Davies (2019:1386), self-definition as a researcher promotes greater research conceptions whilst a narrow conception of research prevented individuals from identifying themselves as active researchers and this may influence postgraduate study progress.

## Conceptual framework

**FIGURE 1:**
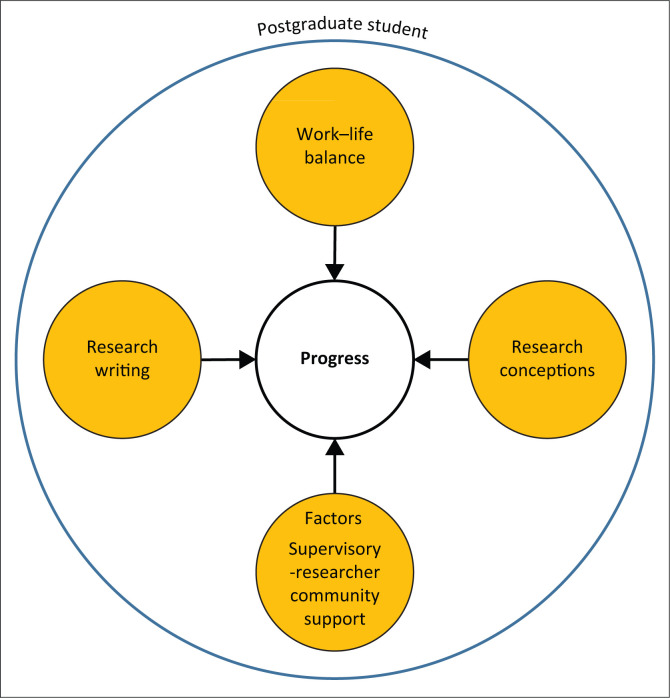
The conceptual framework for the study.

The study’s conceptual framework consisted of core elements that were adopted from the Cross-Country Doctoral Experience Survey (C-DES). The C-DES, developed by Pyhältö et al. (2018:5), was then adapted to suit the Master of Nursing students, with permission from the authors. The C-DES covers seven core elements which include: (1) interest in studies, (2) supervisory-researcher community support, (3) research engagement, (4) burnout, (5) research writing, (6) work–life balance, and (7) research conceptions. However, the manuscript only focuses on four core elements which are: (1) supervisory-researcher community support, (2) research writing, (3) work–life balance, and (4) research conceptions (see Figure 1). The core elements supervisory-researcher community and research writing had a total of 22 items, respectively. The work–life balance had six items, whilst the research conceptions had 10 items. The supervisory-researcher community support had alpha equal to 0.933, research writing 0.803, work–life balance 0.713, and research conceptions 0.726.

The C-DES core elements can be used together or singly (Pyhältö et al. 2018:17). The four core elements from the C-DES are the main stalk from which the various factors that can influence postgraduate students’ study progress arise.

## Problem statement

Most students are excited to begin their postgraduate studies as they may be inspired by friends, lecturers, research experience, and employment opportunities (Guerin, Joyatilaka & Ranasinghe 2015:89). Along the journey, there are however students who fail or progress slowly in their studies, and this has a ripple effect on throughput and dropout rates (Mthimunye, Daniels & Pedro 2018:192; Roos et al. 2016:1). It, therefore, becomes important to understand the perceived factors that Master of nursing students associate with study progress in HEIs. This will enable the determination of the important factors that should be considered in mitigating high attrition, late completions, and poor research outputs of postgraduate students (Lovitts 2008:296). To enable study progress, postgraduate students require continuous supervisory-researcher community support, guidance in research writing, positive research conceptions, and work–life balance (Pyhältö 2018:205).

Statistics reveal that countrywide in South Africa, the overall graduation estimates of master’s students across universities are rock bottom (6.3%) as compared to other countries such as the United Kingdom and Denmark which have 72% and 81%, respectively (European Commission 2015:31; Statistics South Africa 2019:74). There is limited literature that addresses factors such as supervisory-researcher community support, research writing, work–life balance, and research conceptions, and how the Master of Nursing students associate them with study progress. In general, these factors have largely not been explored at the master’s level amongst nursing students.

It is important to establish the factors perceived to be associated with study progress so that remedies could be put in place to create environments where the postgraduate students can flourish academically and in research (Benshoff, Cashwell & Rowell 2015:83). Therefore, the study aimed to determine the perceived supervisory-researcher community support, research writing, work–life balance, and research conceptions factors that Master of Nursing students associated with study progress in selected HEIs in the [Gauteng] province. This study contributes to the body of knowledge on the factors that Master of Nursing students perceive to be associated with study progress which HEIs and nursing departments should strengthen. The study will also enable the nursing departments to develop strategies to address the factors which Master of Nursing students perceived to be negatively associated with study progress.

## Research methodology

### Design

A correlational cross-sectional research design using an adapted questionnaire (Polit & Beck 2017:724) was used.

### Setting

This study was conducted in the nursing departments of three selected HEIs in South Africa. The institutions offer undergraduate academic programmes on a full-time basis and postgraduate academic programmes on a part-time and/or full-time basis, and this include a variety of specialisations at the master’s level.

### Study population and sampling strategy

The study population consisted of the Master of Nursing students who were in their second year of study and subsequent years. The respondents were selected because they had completed at least one year of postgraduate study and therefore they could reflect on factors they perceived and considered important for study progression. The total sampling method was used for recruiting respondents. Postgraduate students were excluded if they had just obtained their Master of Nursing degree. A self-administered questionnaire was sent to all the respondents (*n* = 136) eligible for the study. Completion and submission of the questionnaire to the researcher meant that the respondents consented to participate in the study. The response received was 122 (89.7%).

### Data collection

Data were collected using a multidimensional C-DES self-report questionnaire (Pyhältö et al. 2018:17) with a bio-demographic questionnaire that was attached to the C-DES. The C-DES was originally developed for doctoral students. However, in this study, the C-DES was adapted for the Master of Nursing students after permission was granted by the authors.

The C-DES and bio-demographic questionnaires were e-mailed to the respondents. The bio-demographic questionnaire captured the respondents’ bio-demographic information which included age, gender, marital status, number of children, home language, age, date of birth, and the residential province. The questionnaires were in an editable portable document format (PDF) and the respondents were requested to complete and send them electronically via e-mail to the research representatives from the three HEIs. The research representatives thereafter e-mailed the completed questionnaires to the researcher. The data were captured into a coded data capture Excel sheet by the researcher. Respondents were also e-mailed the information sheet together with the informed consent form. The information sheet contained information on the purpose of the study and informed respondents that participation was voluntary and that no names were to be written on the questionnaires to maintain anonymity. Additionally, it also stated that completion of the questionnaire was regarded as consent to participate in the study. The self-administered questionnaire and information sheet were sent to all eligible respondents by email in the three HEIs. Reminders were sent to all (*n* = 136) respondents three times.

### Data analysis

Descriptive statistics were used at the individual item level to summarise data using percentages and frequencies to identify factors that the postgraduate nursing students associated with study progress. The bio-demographic data were displayed using tables. One item from the supervisory-researcher community support, 11 items from research writing, and three items from work–life balance that were negatively worded were reversed.

Factor analysis was conducted to determine if the items in each of the constructs were effectively grouped. Before the analysis was carried out, the suitability of using factor analysis was assessed using Kaiser-Meyer-Oklin (KMO) and Bartlett’s Test of Sphericity. The requirement is that KMO must be greater than 0.5 and Bartlett’s Test must have a *p*-value less than 0.05 (Napitupulu 2017:697–704).

Diagnostic evaluation, mean standard deviation (s.d.), skewness, and kurtosis were done on the four composite scores of the supervisory-researcher community support, research writing, work–life balance, and research conceptions (see Table 3). Data analysis was done using Statistical Package for the Social Sciences (SPSS) Version 25 (IBM Corp. Released 2017. IBM SPSS Statistics for Windows, Version 25.0. Armonk, NY: IBM Corp. IBM Corp). As the data were ordinal, Spearman’s correlation coefficient was used to measure the relationship amongst the ordinal variables. Ordinal data responses are responses that can be sorted or rank-ordered for example, according to levels of agreement. The Spearman’s correlation coefficient (r_s_) ranges from –1 to +1, where 0 indicates that there is no monotonic association and the relationship gets stronger as *r* approaches absolute 1 (Schober, Boer & Schwarte 2018:1763). Furthermore, if the *p*-value was found to be less than 0.05, it was concluded that the correlation of the two variables was significant.

The Mann-Whitney U test was used to compare the differences between two groups using bio-demographic variables as independent variables. The test assumes that the observations are independent; the dependent variable or response needs to be at least ordinal and the independent variable is assumed to be categorical. The assumption on the homogeneity of variance was tested using Levene’s test of equal variance and it was not met, therefore, results were interpreted in terms of ranks. Where there were more than two groups, the Kruskal-Wallis test was used to check for differences.

### Ethical considerations

Ethical clearance was obtained from the Faculty of Health Sciences Research Ethics Committee of the University of Pretoria (no.: 273/2020). Thereafter, the permission to conduct the study from the other two HEIs was sought before data collection. Completion and submission of the questionnaire to the researcher meant that the respondents consented to voluntarily participate in the study. Respondents were informed about the right to decline to take part in the study without punishment and/or interference with their studies (Beckmann 2017:6). The researcher was not employed at any of the three HEIs. The questionnaires were kept confidential even after data collection. The questionnaire was also coded to ensure respondents’ anonymity.

## Results

The majority of the respondents, 77% (94) were undertaking the Master of Nursing degree part-time. About 81% (99) of the respondents had children. About 55% (67) of respondents were enrolled in the second year of study. Respondents with a source of income were 88.5% (108) and those with no source of income were 11.5% (14) (see Table 1).

**TABLE 1:** Bio-demographic data.

Variables	Frequency	Percentages	Mean age	s.d.
**Age of respondents (*n* = 122)**			40.7	6.7
29–35	33	27.0	-	-
36–40	31	25.4	-	-
41–45	31	25.4	-	-
46+	27	22.1	-	-
**Gender of respondents (*n* = 122)**				
Males	31	25.4	-	-
Females	90	73.8	-	-
Other	1	0.81	-	-
**Marital status (*n* = 122)**				
Married	69	56.6	-	-
Single	30	24.6	-	-
Divorced	14	11.5	-	-
Widowed	1	0.8	-	-
Staying with partner	8	6.6	-	-
**Enrolment status (*n* = 122)**				
Part-time	94	77.0	-	-
Full time	28	23.0	-	-
Dissertation	77	63.1	-	-
Course work and mini-dissertation	45	36.9	-	-
**Children (*n* = 122)**				
No	23	18.9	-	-
Yes	99	81.1	-	-
**Number of children (*n* = 121)**				
0	23	19.0	-	-
1	30	24.8	-	-
2	36	29.8	-	-
3	29	24.0	-	-
4	4	3.3	-	-
6	1	0.8	-	-
**Number of years enrolled (*n* = 122)**				
2	67	54.9	-	-
3	36	29.5	-	-
4	18	14.8	-	-
5	1	0.8	-	-
**Principal source of income (*n* = 122)**				
Respondents with a source of income	108	88.5	-	-
Respondents with no source of income	14	11.5	-	-

s.d., standard deviation.

The distribution of the bio-demographic variables of the study respondents is shown in Table 1.

## Factors that the Master of Nursing students associate with study progress

Exploratory Factor Analysis (EFA) of the factors and individual items that the Master of Nursing students associate with study progress are shown in Table 2. The EFA was done on the Likert 7-point scale. The response levels of the Likert scale were collapsed after the items in the adopted questionnaire were administered to the respondents as 7-point response options.

**TABLE 2:** Frequencies, percentages, and factor loading on the Cross-Country Doctoral Experience Survey questionnaire items.

Factor	Items	Loading	Disagree		Neutral		Agree	
			*n*	%	*n*	%	*n*	%
Supervisory-researcher support (alpha = 0.933) (*n* = 109)	I feel that my supervisors are interested in my opinions.	0.590	15	12.3	11	9.0	96	78.7
	I receive encouragement and personal attention from my supervisors.	0.715	14	11.5	18	14.8	90	73.8
	I feel that my supervisors appreciate my work.	0.738	12	9.8	18	14.8	92	75.4
	I feel that I am treated with respect.	0.762	6	5.0	16	13.3	98	81.7
	My supervisors treat Master’s students in a fair way.	0.749	14	11.5	15	12.3	93	76.2
	I can negotiate central choices regarding my dissertation with my supervisors.	0.753	20	16.7	18	15.0	82	68.3
	I can openly discuss any problems related to my Master’s education with my supervisors.	0.774	18	14.8	11	9.0	93	76.2
	My supervisors express critical comments on my research in a friendly manner.	0.730	14	11.5	7	5.7	101	82.2
	I often receive constructive criticism.	0.734	10	8.3	12	9.9	99	81.8
	I can inform my supervisor if a personal matter affects my work with the dissertation.	0.714	31	25.8	13	10.0	76	63.3
	My supervisors encouraged me to explore alternative viewpoints in my research.	0.726	8	6.6	10	8.2	104	85.2
	My supervisors encourage Master’s students to collaborate with each other.	0.567	26	21.5	24	19.8	71	58.7
	I am satisfied with my supervisor.	0.745	14	11.6	19	15.7	88	72.7
	I feel accepted by my research community.	0.651	17	14.2	19	15.8	84	70.0
	I feel that the other members of my research community appreciate my work.	0.565	20	16.5	24	19.8	77	63.6
	There is a good sense of collegiality amongst the researchers I interact with.	0.509	19	15.7	16	13.2	86	71.1
	I am treated equally in my research community.	0.515	17	14.2	23	18.9	82	67.2
	My research community addresses problems in a constructive way.	0.501	13	10.7	21	17.2	88	72.1
	My expertise is put to use in the research community.	0.621	13	10.7	23	19.5	82	69.5
	I receive encouragement and support from other Master’s students.	0.306	22	18.3	16	13.3	82	68.3
	I can influence issues concerning Master’s education in my research community.	0.424	20	16.4	23	18.9	79	64.8
Research writing (alpha = 0.803) (*n* = 115)	I often postpone research writing tasks until the last moment.	0.641	63	51.6	12	9.8	47	38.5
	I find it difficult to write because I am too critical.	0.372	64	52.5	20	16.4	38	31.1
	My previous writing experiences are mostly negative.	0.389	68	55.7	25	20.5	29	23.8
	Without deadlines, I would not produce anything.	0.482	55	45.1	13	10.7	54	44.3
	I sometimes get completely stuck if I have to produce texts.	0.640	46	37.7	20	16.4	56	45.9
	I find it difficult to start writing.	0.679	56	45.9	14	11.5	52	42.6
	I find it easier to express myself in other ways than writing.	0.397	40	34.2	25	21.4	52	44.4
	The skill of writing is something we are born with; all of us cannot learn it.	0.465	86	70.5	19	15.6	17	13.9
	I find it difficult to hand over my texts because they never seem complete.	0.634	64	52.5	15	12.3	43	35.2
	I start writing only if it is absolutely necessary.	0.638	55	45.1	15	12.3	52	42.6
	I hate writing.	0.526	73	59.8	18	14.8	31	25.4
Work–life balance (alpha = 0.713) (*n* = 122)	I neglect my personal needs because of my studies.	0.407	45	35.3	18	7.4	70	42.7
	I am too tired to be effective in my studies.	0.478	55	45	20	16.4	47	38.6
	My job makes my study life difficult.	0.476	47	38.5	15	12.3	60	49.2
Research conceptions (alpha = 0.726) (*n* = 118)	Doing research is a matter of personal development.	0.526	3	2.5	0	0	119	97.5
	Doing research is a matter of personal learning and growth.	0.554	2	1.6	1	0.8	119	97.5
	Doing research has to do with developing a personal journey.	0.517	3	2.5	7	5.8	110	91.7
	Doing research has to do with being famous or recognised in your area of work.	0.448	50	41.3	25	20.7	46	38.0
	Doing research has to do with having your papers published and others reading them.	0.377	26	21.3	18	14.8	78	63.9
	Doing research has to do with obtaining qualifications and gaining accomplishments.	0.349	22	18.0	12	9.8	88	72.1
	Doing research has to do with extending current concepts to obtain a better understanding.	0.570	3	2.5	3	2.5	116	95.1
	Doing research has to do with finding solutions to the problems.	0.628	0	0.0	4	3.3	118	96.7
	Doing research has to do with answering questions.	0.469	4	3.3	5	4.1	112	92.6
	Doing research has to do with providing deeper insight and understanding of a particular topic.	0.679	0	0.0	3	2.5	119	97.5

The suitability of the data set for EFA was tested using a KMO test. A higher KMO indicates sampling adequacy for each model variable, with values > 0.8 being ideal (Feng et al. 2017:1140). Bartlett’s test of sphericity performed showed that the item set were correlated as *p* < 0.05. This method was used in conjunction with scree plot to visualise the proposed factors and their relative eigenvalues. Factors that had eigenvalues greater than one were retained. The extraction method used was principal component analysis with rotation method Varimax with Kaiser Normalisation. The rotated solution showed the presence of a simple structure with all components showing a number of strong loadings and all variables loading substantially on only one component. The items were grouped into four factors as detailed earlier. The reliability of the EFA model was determined by calculating Cronbach’s alpha which provides a measure of internal consistency for each factor.

Exploratory Factor Analysis performed with the factors that the Masters of Nursing students associate with study progress variables suggested that the four components with eigenvalues exceeding 1 must be retained explaining 43% of the variance (see Table 2) (Amerioun et al. 2018:1522). Component one explained 20.6% of the variance, component two 10%, component three 7.1%, and component four 5.6%. Cronbach Alpha for the factors were calculated (excluding the missing values) with supervisory-researcher community support having an alpha equal to 0.933 (*n* = 109), research writing 0.803 (*n* = 115), work–life balance 0.713 (*n* = 122) and research conceptions 0.726 (*n* = 118). These findings are consistent with those of the original C-DES questionnaire (Pyhältö et al. 2018:10–17). All the items that were loaded onto each respective component were also presented (see Table 2). The item ‘I feel like an outsider in my own research community’ was excluded from the supervisor-researcher community support based on low factor loading. All 22 items loaded strongly on component one (supervisory-researcher community support) except only one item (I feel like an outsider in my own research community) which had a coefficient of 0.24 and the rest had > 0.3. On component two (research writing perceptions), 11 items were loaded strongly with coefficients of > 0.37 and the other 11 items had < 0.3. On work–life balance, three out of six items were retained. The ones which were excluded were: ‘I am able to combine my career and life goals such as the desire for children’, ‘my work as a researcher is in line with my personal values’ and ‘I am satisfied with my work–life balance’. All three items were loaded strongly on component three (work–life balance) with coefficients of > 0.4 and the rest had coefficients of < 0.3. All 10 items on research conceptions (component four) were loaded strongly with coefficients of > 0.35. Additionally, Table 2 presents summarised responses which were grouped into disagree, neutral, and agree. The strongly agree, partially agree, and agree categories were grouped into the agree category. The neutral category was not grouped. The strongly disagree, partially disagree, and disagree categories were grouped into the disagree category. In Table 2, the Likert scale was collapsed to allow greater clarity and identification of trends or patterns in the responses (Beamish [2004] and DeVaus [1995] in DiStefano, Shi & Morgan [2020:237]).

## Composite scores of the core elements

The composite scores of responses of the perceived factors that the Master of Nursing students associate with study progress were calculated from the responses of each respondent on a 7-point Likert scale to determine the average scores of each of the four core elements for each respondent. Missing values were left out in the calculations of the mean and the sample for analysis was reduced by the number of missing values per item. Diagnostic evaluation was done on the four composite scores that were derived and the standard deviation, skewness and kurtosis results, respectively, were as follows: supervisory-researcher community support (1.155; −0.651; 0.012), research writing perceptions (1.074; 0.054; −0.011), work–life balance (1.452; −0.169; −0.192), and research conceptions (0.736; −0.614; 0.317). The skewness was identified in data on the composite scores. A higher mean composite score in any of the four composite scores indicated greater experience of each of the core elements, for example, a higher score on supervisory-research community support indicates that respondents experienced or perceived greater supervisory-research community support. The above findings, presented in Table 3, show that all the variables except research writing were negatively skewed. The results of the calculations showed that the mean response for supervisory-researcher community support was 5.338 with a s.d. of 1.155. Research writing had a mean response of 3.667 and s.d. of 1.074. The work–life balance had a mean response of 4.134 and s.d. of 1.452. Research conceptions had a mean response of 5.729 and s.d. of 0.736.

**TABLE 3:** Descriptive statistics of composite scores.

Core elements	*n*	Statistic					
		Minimum	Maximum	Mean	s.d.	Skewness	Kurtosis
Supervisory-researcher community support	109	1.00	7.00	5.338	1.155	−0.651	0.012
Research writing perceptions	115	1.00	7.00	3.667	1.074	0.054	−0.011
Work–life balance	122	1.00	7.00	4.134	1.452	−0.169	−0.192
Research conceptions	118	3.00	7.00	5.729	0.736	−0.614	0.317

s.d., standard deviation.

## Supervisory-researcher community support results

Considering the individual items of the supervisory-researcher community support core element, most researchers 81.7% (98) agreed to being treated with respect by their supervisors whilst 5% (six) disagreed and 13.3% (16) remained neutral. The majority of respondents 82.2% (101) agreed that their supervisors expressed critical remarks on their research in an approachable manner, whilst 11.5% (14) disagreed and 5.7% (seven) were neutral. About 85% (104) of the study respondents indicated that their supervisors encouraged them to explore alternative viewpoints in their research, whilst 6.6% (eight) disagreed. Additionally, the majority 81.8% (99) of the respondents stated that they often received constructive criticism, whilst 8.3% (10) disagreed. Respondents who disagreed with being encouraged to collaborate with other master’s students were 21.5% (26) and those who agreed were 58.7% (71). Furthermore, about 26% (31) of the respondents disagreed that they were able to inform their supervisors if a personal issue affected their dissertation related-work, however, 10% (13) remained neutral and 63.3% (76) agreed. Few respondents 18.3% (22) indicated that they received encouragement and support from other master’s students whilst 68.3% (82) agreed with the notion (see Table 2).

**FIGURE 2:**
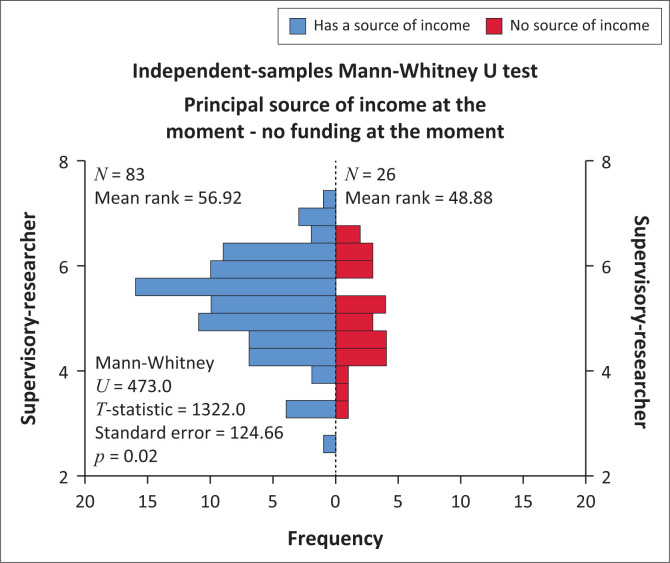
Comparison of supervisory-researcher community support scores between the groups with and without a source of income.

The Mann-Whitney U test was carried out to compare the supervisory-researcher community support scores between the groups with and without a source of income. The sample size of the two groups indicates this as n1 = 83 (with income) versus n2 = 26 (without income). The distribution of the two groups on the supervisory-researcher community support score had different shapes therefore, mean ranks were used to compare the groups (Levene’s test of equal variances: F(df) = 3.56, *p* = 0.043). Respondents with a source of income (mean rank = 56.92) differed in supervisory-researcher community support from respondents without income (mean rank = 48.88, U = 473.0, *p* = 0.02) (see Figure 2), they perceived greater supervisory-researcher community support than those with no source of income. Respondents who were studying part-time (mean rank = 58.27) did not differ in supervisory-researcher community support perceptions from those studying full time (mean rank = 56.96), U = 1067, *p* = 0.863.

## Research writing perceptions results

Considering the individual items of the research writing perceptions, slightly more than half of the respondents 52% (63) disagreed that they often postpone writing tasks to the last moment, with 10% (12) were neutral. About 44.3% (54) of the respondents agreed that without deadlines, they will not produce anything whilst 45.1% (55) disagreed and 20.5% (25) remained neutral. More respondents 52.5% (64) disagreed compared to 31.1% (38) who agreed that they found it difficult to write because they are too critical. Most respondents 44.4% (52) agreed to finding it easier to express themselves in other ways than writing whilst 34.2% disagreed and 21.4% (25) were neutral. More respondents 45.9% (56) agreed to sometimes getting completely stuck if they had to produce texts compared to 37.7% (46) who disagreed and 16.4% were neutral. There was an almost even split in respondents who agreed 44.3% (54) and those who disagreed 45.1% (55) to not producing any work without deadlines.

**FIGURE 3:**
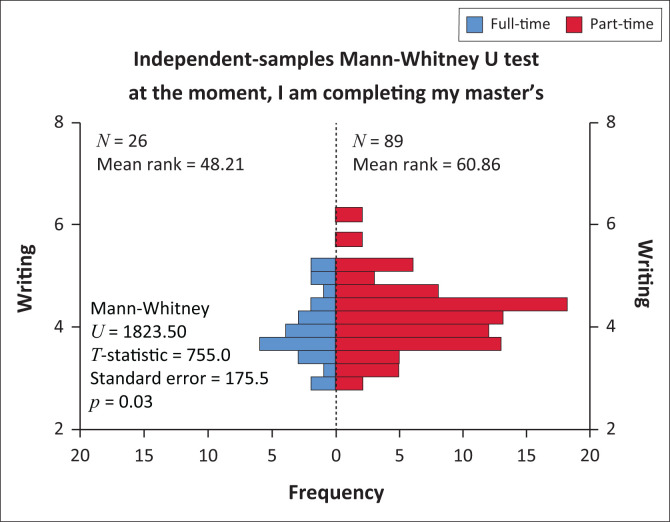
The comparison of perceptions of part-time and full-time Master of Nursing students regarding research writing using the Mann-Whitney U test.

The Mann-Whitney U test was carried out to compare the research writing scores between the groups of those studying full-time and part-time. The sample size of the two groups indicates this as n1 = 26 (full-time) versus n2 = 89 (part-time). The distributions of the two groups on the research writing score had different shapes therefore mean ranks were used to compare the groups (Levene’s test of equal variances indicated unequal variances with F(df) = 4.56, *p* = 0.003). Respondents studying full-time (mean rank= 48.21) differed in research writing from respondents studying part-time (mean rank = 60.86), U = 1823.50, *p* = 0.03 as shown in Figure 3. Respondents who were studying full-time perceived better support in research writing than those studying part-time. However, the research writing scores between the groups of respondents with income and those without income showed no statistical difference. Respondents with income (mean rank = 61.67) were not different in research writing perception from those without income (mean rank = 61.61), U = 1370.0, *p* = 0.953.

## The work–life balance factors’ results

Considering individual items of the work–life balance, about 42.7% (70) respondents agreed to neglect personal needs because of studies whilst 35.3% (45) disagreed and 7.4% were neutral. Respondents who indicated that they were too tired to be effective in their studies were 38.6% (47) whilst 45% (55) disagreed with the notion and 16.4% (20) remained neutral. About 49% (60) of the respondents indicated that their job made their study life difficult whilst 38.5% (47) disagreed and 12.3% (15) remained neutral.

The Mann-Whitney U test was carried out to compare the work–life balance scores between the groups of those studying full-time and part-time. Levene’s test of equal variances was F(df) = 2.45, *p* = 0.033. Respondents studying full-time (mean rank = 58.20) were not significantly different in work–life balance perceptions from respondents studying part-time (mean rank = 62.48), U = 1223.50, *p* = 0.572. Respondents with income (mean rank = 58.44) were not significantly different in work–life balance perceptions from respondents studying part-time (mean rank = 70.88), U = 1661, *p* = 0.093.

## The research conceptions results

Considering the individual items of the research conceptions, about 98% (119) of the respondents agreed that conducting research was a matter of personal development, personal learning, and growth and provided deeper insight and understanding of a particular topic. Nevertheless, 41.3% (50) disagreed that conducting research has to do with being famous or being recognised in one’s area of work whilst 38% (46) agreed. Additionally, 63.9% (78) of the respondents agreed to perceiving research as having one’s papers published and others reading them, whilst 21.3% (26) disagreed and 14.8% (18) remained neutral. About 72% (88) of the respondents indicated that conducting research had to do with obtaining qualifications and gaining accomplishments. However, 18% (22) disagreed and 9.8% (12) were neutral. About 95% (116) of the respondents regarded conducting research as extending current concepts to obtain a better understanding whilst 2.5% (3) agreed and the other 2.5% (3) remained neutral. Respondents who agreed that research has to do with finding solutions to problems were 96.7% (118) whilst those who disagreed were 3.3% (4). About 93% (112) of the respondents perceived it as having to do with answering research questions whereas 3.3% (4) disagreed and 4.1% (5) were neutral.

The Mann-Whitney U test was carried out to compare the research conceptions scores between the groups of those studying full-time and part-time. Levene’s test of equal variances indicated unequal variances of F(df) = 4.20, *p* = 0.001. Respondents studying full-time (mean rank = 63.59) were not significantly different in research conceptions perceptions from respondents studying part-time (mean rank = 60.88), U = 1257.50, *p* = 0.721. The Mann-Whitney U test was also carried out to compare the research conceptions scores between the groups of those respondents with income and those without. Levene’s test of equal variances indicated unequal variances of F(df) = 3.66, *p* = 0.012. The Mann-Whitney U test results showed there was no statistical difference between respondents with income (mean rank = 65.68) and those without income (mean rank = 60.14), U = 1254.50 had a *p*-value of 0.455.

## The Spearman’s correlation between the four core elements

**FIGURE 4:**
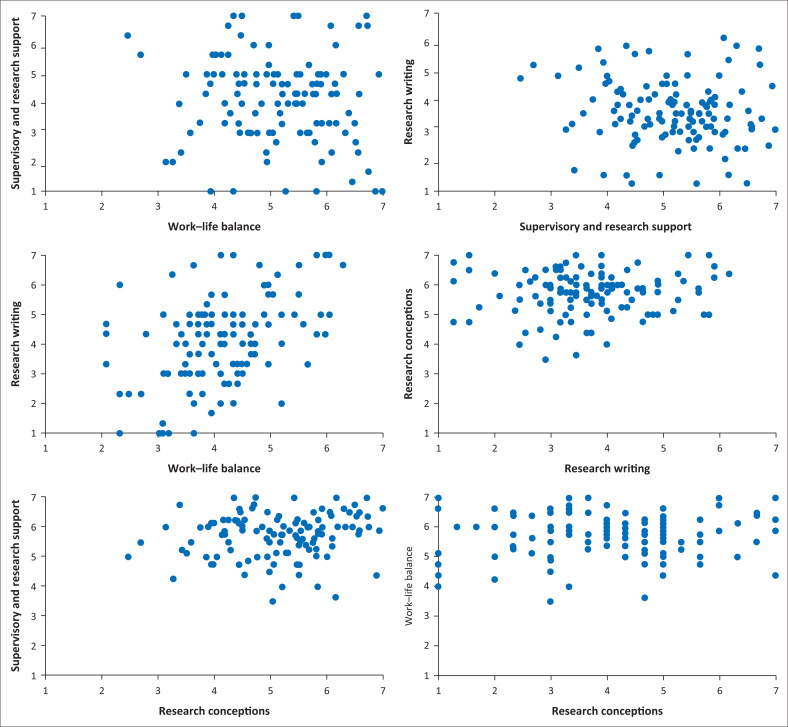
The scatterplots showing the relationship between the factors that Master of Nursing students associate with study progress.

Spearman’s Correlation test analysis was done between the core elements of this study. There was a low positive correlation between supervisory-researcher community support and research conceptions (*r*_s_ = 0.179; *p* = 0.048) and work–life balance and research writing (*r*_s_ = 0.394; *p* < 0.001). The rest of the correlations were not statistically significant: research conceptions and research writing (*r*_s_ = 0.016; *p* = 0.856), work–life balance and research conceptions (*r*_s_ = 0.045; *p* = 0.622), supervisory-researcher community support and work–life balance (*r*_s_ = −0.119; *p* = 0.193), and supervisory-researcher community support and research writing (*r*_s_ = −0.160; *p* = 0.078). These findings are further demonstrated in the scatter plots in Figure 4.

The Scatter plots presented in Figure 4 show that there was a low positive correlation between supervisory-researcher community support and research conceptions. A low positive correlation was also noted between work–life balance and research writing perceptions, this suggested that the more the students managed their work–life balance, the better was their research writing perceptions. The rest of the correlations were not statistically significant.

Spearman’s correlation was carried out to evaluate the relationship between core elements and age. The results show that there was a weak relationship of age with supervisory-researcher community support (*r*_s_ = 0.106), research writing perceptions (*r*_s_ = −0.062), work–life balance (*r*_s_ = −0.013), and research conceptions (*r*_s_ = 0.029).

## Kruskal-Wallis Test on the effect of age on the core elements

The Kruskal-Wallis test results showed that the younger age group experienced greater supervisory-researcher community support with a mean rank of 83.5, whereas the 41–50 age group experience less support. Research conceptions were less pronounced in the older group of 41–50 and 50 and above (mean rank 63.39 and 67.0, respectively). Overall age had no significant effect on the responses given on supervisory-researcher community support and research writing because there was no substantial difference in the ranks amongst the groups.

## Discussion

In this study, the supervisory-researcher community support had a high mean composite score indicating that Master of Nursing students perceived greater supervisory-researcher community support. This notion was supported by Van Wyk et al. (2016:16) and Kaakinen et al. (2016:22) who asserted that supervisory-research support in the form of peer-support received increased opportunities for learning thus promoting study progress.

Taking into consideration the individual item level of the supervisory-researcher support, being treated with respect by supervisors was valued by most respondents. This notion was supported by Van Wyk et al. (2016:26) who reiterated that the existence of trust between the postgraduate student and the supervisor had a positive influence on the Master of Nursing students’ study progress. This feel-good factor can positively influence study progress.

Most of the respondents perceived their supervisors to exhibit the ability to express critical remarks on their research in a friendly manner. This is in agreement with Ali, Watson and Dhingra’s (2016:227) study which indicated that postgraduate students expected their supervisors to provide timely and constructive feedback and to be friendly and approachable.

Master of Nursing students who had a source of income perceived greater supervisory support than those with no source of income. These findings were supported by Bolli, Agasisti and Johnes (2015:396). Bolli et al. (2015:396) reiterated that lack of funding and the use of personal earnings contributed towards delayed study progress and completion. Funding is critical as postgraduate students are faced with difficulties in paying tuition fees, transport, and research-related expenses. However, students with funding only need to concentrate on their studies which may contribute towards good study progression to a greater extent. The pool of funding available for postgraduate students is very little and may need to be increased to assist postgraduate nursing students with funding opportunities

The Kruskal-Wallis test results showed that the 30 years and younger age group experienced greater supervisory-researcher community support with a mean rank of 83.5 whereas the 41–50 age group experienced less. There is a dearth of literature that supports these above-mentioned study results; however, the 30 years and younger age group could have experienced greater supervisory-researcher community support than the older age group possibly because maybe the older age is more able to work independently without the need for more support from their supervisors.

At the composite level, there were divided perceptions amongst students regarding the support they received in research writing. This meant that some Master of Nursing students perceived greater support in research writing whilst some perceived less support.

Some Master of Nursing students exhibited perfectionism traits at the individual item level, as they revised texts endlessly until they completely got stuck when they had to produce text, in an attempt to make their dissertation perfect (Pyhältö et al. 2018:14–15) resulting in impeded study progress. Respondents indicated that they sometimes completely got stuck when they had to produce texts. This inability to produce texts indicated that the respondents experienced writing blocks that impeded progress (Pyhältö et al. 2018:14). In the same vein, respondents stated that they found it easier to express themselves by other means rather than writing. It is clear from these findings that writing skills were needed and perceived to be of importance by most postgraduate students registered for the Master of Nursing degree in this cohort. There was an almost even split between students who agreed to not producing any work without deadlines and those who disagreed which is a cause for concern. The even split may suggest that supervisors should check their postgraduate students’ progress and also try to come up with the best strategy for postgraduate students to use regarding meeting dissertation milestones deadlines. This will aid postgraduate student study progress.

Master of Nursing students who were studying full-time self-reported greater support in research writing than those studying part-time. These results concur with the findings from Lowe and Gayle (2007:225) who indicated full-time students studied for 30 h on average and 16–20 h were spent on working. On the other hand, part-time students spent 10 h and 40 h per week studying and working, respectively. This resulted in the differences in the levels of study progression as the former (full-time students) had more study time than the latter (part-time students). It would be of interest to check the differences in completion rates between those postgraduate students studying full-time against those studying part-time.

A fairly high mean composite score on work–life balance meant that the students perceived considerable support in work–life balance. Despite this fairly high mean composite score on work–life balance, at the individual item level, some respondents found it difficult to balance work and life as they indicated that they neglected their personal needs because of studies. The neglect of personal needs by the study respondents was also established by Kennett, Reed and Van den Berg (2019:138) who indicated that postgraduate students who spent less time on academic activities and more on personal needs such as leisure had lesser perceptions of their ability to balance academic and non-academic tasks. The differences in the perceptions could be attributed to failure to balance work–life commitments and possibly lack of social support that could have contributed to the neglect of personal needs because of studies thus impacting study progress negatively.

About 49% (60) of respondents alluded that their job made their study life difficult. This finding at individual item level findings was supported by Benshoff et al. (2015:83) who indicated that the multiple roles, responsibilities, and expectations impeded postgraduates’ study progress. Moreover, some postgraduate students reiterated that their jobs required them to travel a lot leaving them with inadequate time to focus on their thesis resulting in a delayed study thesis schedule (Benshoff et al. 2015:83). Therefore, there should be open communication between the supervisor and the postgraduate student so that the student may feel comfortable to openly discuss their work–life balance thus promoting study progress.

There is a dearth in the literature that indicates that Master of Nursing students perceived that work–life led to better writing perceptions. It may be possible that those with a balanced work–life balance may have time to focus on research writing.

The research conceptions core elements had a high mean composite score. The findings are in line with Ross et al. (2017:73) whose study indicated that the majority 48 h (52%) of postgraduate experienced positive research conceptions. Cera, Cristini and Antonietti (2018:241)’s study showed that to a large extent, older respondents’ had conceptions of learning as an interpersonal and focused process. These results are in line with the study results which showed that respondents who were 50 years and older had greater perceptions of the research conception core element.

Considering individual items of research concepts, most respondents (95%) indicated that doing research included extending current concepts to obtain a better understanding. This was further cemented by the finding that 97.5% of the cohort agreed that the purpose of doing research had to do with providing deeper insight and understanding of a particular topic. These results are aligned with findings from studies conducted by Pyhältö et al. (2018:17), Ekpoh (2016:67) and Ross et al. (2017:73), who agree that the above-mentioned research conceptions contributed positively to postgraduate students’ study progress. An overwhelming number (97.5%) of the respondents perceived research as a matter of personal development, learning and growth. These results are supported by studies conducted by Pyhältö et al. (2018:16–17) and Ekpoh (2016:67) whose common themes reflected that conceiving research as personal development, learning and growth had a positive impact and promoted thesis/dissertation progress.

There was a positive correlation between work–life balance and research writing (*r* = 0.394; *p* < 0.001). The study showed that greater work–life balance was perceived by the respondents to be positively associated with better research writing perceptions. This may be because of postgraduate students being able to manage their time between work–life and studies (Martinez et al. 2013:39) and thus freeing time for research writing which then enables study progression.

The study finding indicated that there was a low positive correlation between supervisory-researcher community support and research conceptions. There is however a deficit in literature to support these results. The low positive correlation could have been because of the respondents perceiving supervisory-researcher community support to be less associated with research conceptions.

Additionally, a low positive correlation was also noted between research writing and work–life balance. There is a lack of literature that supports this notion. However, it may be possible that the respondents who perceived greater support in research writing did not associate it with a balanced work–life.

## Strengths

Knowledge of supervisory-researcher community support and research writing perceptions may aid institutions to develop policies and teaching and learning strategies that will aid Master of Nursing students to progress and complete their studies in time. The study findings may contribute to providing clarity on the perceived factors that Master of Nursing students associate with study progress which HEIs and nursing departments should strengthen. The study may also enable the nursing departments to develop strategies to address the factors that Master of Nursing students perceive less support in. Additionally, the study may contribute to the body of knowledge that seeks to explore literature relating to the progress of Master of Nursing students, thus spurring research in other related areas nationally and internationally.

## Limitations

The study respondents were obtained from only three HEIs in one province which may limit the generalisation of the findings to other HEIs in other provinces of South Africa. In addition, the study was also confined to Master of Nursing students, therefore the study findings cannot be generalised to other postgraduate students such as those undertaking Honours and PhD studies. Furthermore, the study was not able to explicitly measure study progression and investigate the relationship between the C-DES factors and study progression.

## Recommendations

This study recommends that HEIs and Department of Higher Education and Training (DHET) policymakers should look into postgraduate student funding as the findings from the study implied that Master of Nursing students who had a source of income perceived greater supervisory-researcher community support than those who had no funding. The HEIs should consider providing research writing support in the form of writing retreats and workshops so that Master of Nursing who experience blocks and procrastinators may benefit. Recommendations for future research are that a longitudinal study or a retrospective study looking at throughput and graduation rates of Master of Nursing students may be looked into.

## Conclusion

The study revealed that Master of Nursing students who had a source of income perceived greater supervisory-researcher community support than those who had no funding. Additionally, students who were studying full-time perceived greater research writing support than those who were studying part-time. At the individual item level, some Master of Nursing students perceived challenges with research writing perceptions and some aspects of work–life balance such as their jobs making their study life difficult. Adequate supervisory-researcher community support was greatly perceived as contributing to improved research writing perceptions which may aid study progress, throughput, and completion rates. The results of the study also reflected that despite the study respondents largely having greater perceptions towards supervisory-researcher community support, respondents who were 30 years and below age experienced more supervisory-researcher community support than the older group of 41–50 years. Furthermore, most respondents at the composite level had greater perceptions regarding the research conception core element. The study adds to the available body of literature on the factors that Master of Nursing students associate with study progress. The study may act as a springboard for further related studies.
